# 4-(2-Hydroxy­ethyl)anilinium 3,5-dinitro­benzoate

**DOI:** 10.1107/S1600536809030426

**Published:** 2009-08-08

**Authors:** Graham Smith, Urs D. Wermuth

**Affiliations:** a School of Physical and Chemical Sciences, Queensland University of Technology, GPO Box 2434, Brisbane, Qld 4001, Australia

## Abstract

In the title compound, C_8_H_12_NO^+^·C_7_H_3_N_2_O_6_^−^, the anilinium and hydroxyl protons of the cation result in N—H⋯O, N—H⋯(O,O) and O—H⋯O hydrogen-bonding inter­actions with carboxyl­ate O-atom acceptors, forming a two-dimensional network structure.  An intermolecular C—H⋯O interaction is also present.

## Related literature

For related structures, see: Etter & Frankenbach (1989 ▶); Lynch *et al.* (1991*a* ▶,*b* ▶, 1992 ▶, 1993 ▶); Ranganathan & Pedireddi (1998 ▶); Aakeröy *et al.* (2003 ▶); Hosomi *et al.* (2000 ▶).
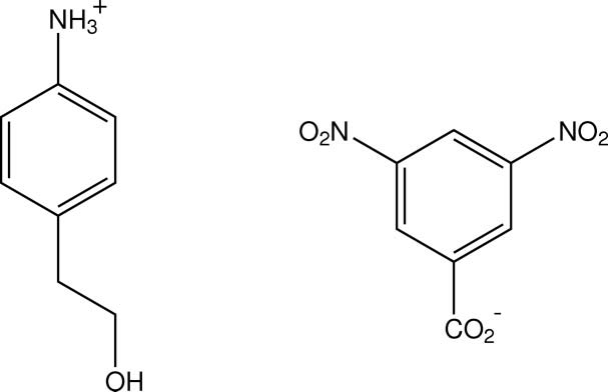


## Experimental

### 

#### Crystal data

C_8_H_12_NO^+^·C_7_H_3_N_2_O_6_^−^
*M* *_r_* = 349.30
Monoclinic, 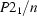

*a* = 15.9566 (19) Å
*b* = 5.7844 (5) Å
*c* = 17.4118 (14) Å
β = 102.811 (10)°
*V* = 1567.1 (3) Å^3^
*Z* = 4
Mo *K*α radiation
μ = 0.12 mm^−1^
*T* = 297 K
0.30 × 0.30 × 0.25 mm

#### Data collection

Oxford Diffraction Gemini-S CCD-detector diffractometer
Absorption correction: multi-scan (**SADABS**; Sheldrick, 1996 ▶) *T*_min_ = 0.950, *T*_max_ = 0.980
5928 measured reflections
3061 independent reflections
2203 reflections with *I* > 2σ(*I*)
*R*_int_ = 0.017

#### Refinement

*R*[*F* ^2^ > 2σ(*F* ^2^)] = 0.037
*wR*(*F* ^2^) = 0.099
*S* = 0.98
3061 reflections
242 parameters
H atoms treated by a mixture of independent and constrained refinement
Δρ_max_ = 0.21 e Å^−3^
Δρ_min_ = −0.17 e Å^−3^

### 

Data collection: *CrysAlis Pro* (Oxford Diffraction, 2009 ▶); cell refinement: *CrysAlis Pro*; data reduction: *CrysAlis Pro*; program(s) used to solve structure: *SIR92* (Altomare *et al.*, 1994 ▶); program(s) used to refine structure: *SHELXL97* (Sheldrick, 2008 ▶); molecular graphics: *PLATON* (Spek, 2009 ▶); software used to prepare material for publication: *PLATON*.

## Supplementary Material

Crystal structure: contains datablocks global, I. DOI: 10.1107/S1600536809030426/bt5012sup1.cif

Structure factors: contains datablocks I. DOI: 10.1107/S1600536809030426/bt5012Isup2.hkl

Additional supplementary materials:  crystallographic information; 3D view; checkCIF report

## Figures and Tables

**Table 1:** Hydrogen-bond geometry (Å, °)

*D*—H⋯*A*	*D*—H	H⋯*A*	*D*⋯*A*	*D*—H⋯*A*
O11*A*—H11*A*⋯O11^i^	0.89 (2)	1.88 (2)	2.7569 (16)	168 (2)
N4*A*—H41*A*⋯O12	0.958 (19)	1.924 (19)	2.845 (2)	160.8 (17)
N4*A*—H42*A*⋯O11^ii^	0.936 (19)	2.02 (2)	2.8905 (19)	154.0 (18)
N4*A*—H42*A*⋯O12^ii^	0.936 (19)	2.53 (2)	3.1033 (18)	119.9 (14)
N4*A*—H43*A*⋯O11*A*^iii^	1.005 (19)	1.783 (19)	2.785 (2)	174.4 (19)
C5*A*—H5*A*⋯O11*A*^iv^	0.93	2.43	3.317 (2)	161

Symmetry codes: (i) 
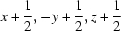
; (ii) 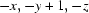; (iii) 
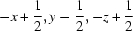
; (iv) 
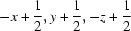
.

**Fig. 1.**
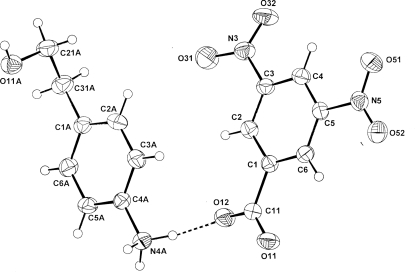
Molecular configuration and atom naming scheme for the substituted anilinium cation and the 3,5-dinitrobenzoate anion in (I). Displacement ellipsoids are drawn at the 50% probability level.

**Fig. 2.**
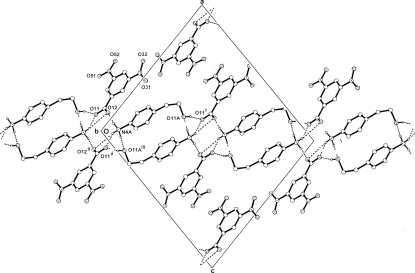
The two-dimensional hydrogen-bonded network structure of (I) extending across the (a0c) plane and viewed down the approximate b axial direction of the unit cell, showing hydrogen-bonding associations as dashed lines. Non-interactive H atoms are omitted. For symmetry codes, see Table 1.

## supplementary crystallographic information

### Comment

The nitro-substituted aromatic acid, 3,5-dinitrobenzoic acid (3,5-DNBA) has been used to synthesize chiral crystalline adduct materials with physical properties potentially useful in applications such as nonlinear optics, giving *e.g.* the compound 3,5-DNBA–4-aminobenzoic acid (1/1) (Etter & Frankenbach, 1989). Since that time there have been a large number of 3,5-DNBA adduct structures reported, *e.g.* with indole-3-acetic acid (1:1) (Lynch *et al.*, 1991*a*), phenoxyacetic acid (a 2:1 monohydrate) (Lynch *et al.*, 1991*b*), 1,4-diiodobenzene (2:1) (Ranganathan & Pedireddi (1998)], a series of alkyl-substituted carbazoles (all 1:1) (Hosomi *et al.*, 2000) and benzamide (1:1)] (Aakeröy *et al.*, 2003); Proton-transfer compounds and proton-transfer-3,5-DNBA adduct compounds are also very common, *e.g.* with the herbicides amitrole (3-amino-1,2,4-triazole)  and prometryn (*N,N*'-bis(1-methylethyl)-6- methylthio-1,2,4-triazine-2,4-diamide) (Lynch *et al.*, 1993) (all 1:1)

In the light of this background we looked at 3,5-DNBA as a possible means of obtaining a crystalline compound from the non-crystalline aromatic Lewis base 2-(4-aminophenyl)ethanol. The 1:1 stoichiometric reaction of 3,5-DNBA with this reagent in 50% ethanol–water was expected to give either an anilinium salt or an adduct salt and the result was a 1:1 salt 4-(2-hydroxyethyl)anilinium 3,5-dinitrobenzoate C_8_H_12_NO^+^. C_7_H_3_N_2_O_6_^-^ (I), the structure of which is reported here.

With (I) (Fig. 1), proton transfer occurs and the resulting anilinium group is subsequently involved in four hydrogen-bonding interactions with only carboxylate-O acceptors (Table 1). One of these associations is asymmetric cyclic [N–H···O,*O*', graph set *R*^2^_1_(4)]. These interactions, together with an hydroxyl *O*–*H*···O_carboxyl_ hydrogen bond give a two-dimensional network structure which lies in the (*a*0*c*) plane and extends down the *b* cell direction (Fig. 2). Also present in the structure are short inversion-related intermolecular nitro O···O nonbonding interactions [O32···O32^iv^, 2.8799 (18) Å; symmetry code: (iv) -*x* + 1, -*y*, -*z*]. The 3,5-DNBA anion is essentially planar [C2–C1–C11–O11A, -172.71 (13)° (carboxyl); C2–C3–N3–O32, -161.49 (14)° and C4–C5–N5–O52, -177.25 (13)° (nitro)].

### Experimental

The title compound was synthesized by heating together 1 mmol quantities of 2-(4-aminophenyl)ethanol with 3,5-dinitrobenzoic acid in 50 ml of 50% ethanol–water under reflux for 10 minutes. After concentration to *ca* 30 ml, partial room temperature evaporation of the hot-filtered solution gave light brown coloured flat prisms (m.p. 389 K).

### Refinement

Hydrogen atoms involved in hydrogen-bonding interactions were located by difference methods and their positional and isotropic displacement parameters were refined. The H-atoms bonded to C were included in the refinement in calculated positions [C–H(aliphatic) = 0.97 Å and C–H(aromatic) = 0.93 Å) using a riding model approximation, with *U*_iso_(H) = 1.2*U*_eq_(C).

### Crystal data

**Table:**

C_8_H_12_NO^+^·C_7_H_3_N_2_O_6_^−^	*F*(000) = 728
*M**_r_* = 349.30	*D*_x_ = 1.480 Mg m^−^^3^
Monoclinic, *P*2_1_/*n*	Melting point: 389 K
Hall symbol: -P 2yn	Mo *K*α radiation, λ = 0.71073 Å
*a* = 15.9566 (19) Å	Cell parameters from 3103 reflections
*b* = 5.7844 (5) Å	θ = 3.0–28.9°
*c* = 17.4118 (14) Å	µ = 0.12 mm^−^^1^
β = 102.811 (10)°	*T* = 297 K
*V* = 1567.1 (3) Å^3^	Cut block, pale brown
*Z* = 4	0.30 × 0.30 × 0.25 mm

### Data collection

**Table:**

Oxford Diffraction Gemini-S CCD-detector diffractometer	3061 independent reflections
Radiation source: Enhance (Mo) X-ray source	2203 reflections with *I* > 2σ(*I*)
graphite	*R*_int_ = 0.017
ω scans	θ_max_ = 26.0°, θ_min_ = 3.6°
Absorption correction: multi-scan (*SADABS*; Sheldrick, 1996)	*h* = −19→19
*T*_min_ = 0.950, *T*_max_ = 0.980	*k* = −4→7
5928 measured reflections	*l* = −21→15

### Refinement

**Table:**

Refinement on *F*^2^	Primary atom site location: structure-invariant direct methods
Least-squares matrix: full	Secondary atom site location: difference Fourier map
*R*[*F*^2^ > 2σ(*F*^2^)] = 0.037	Hydrogen site location: inferred from neighbouring sites
*wR*(*F*^2^) = 0.099	H atoms treated by a mixture of independent and constrained refinement
*S* = 0.98	*w* = 1/[σ^2^(*F*_o_^2^) + (0.0603*P*)^2^] where *P* = (*F*_o_^2^ + 2*F*_c_^2^)/3
3061 reflections	(Δ/σ)_max_ < 0.001
242 parameters	Δρ_max_ = 0.21 e Å^−^^3^
0 restraints	Δρ_min_ = −0.17 e Å^−^^3^

### Special details

**Table:**

Geometry. Bond distances, angles *etc*. have been calculated using the rounded fractional coordinates. All su's are estimated from the variances of the (full) variance-covariance matrix. The cell e.s.d.'s are taken into account in the estimation of distances, angles and torsion angles
Refinement. Refinement of *F*^2^ against ALL reflections. The weighted *R*-factor *wR* and goodness of fit *S* are based on *F*^2^, conventional *R*-factors *R* are based on *F*, with *F* set to zero for negative *F*^2^. The threshold expression of *F*^2^ > σ(*F*^2^) is used only for calculating *R*-factors(gt) *etc*. and is not relevant to the choice of reflections for refinement. *R*-factors based on *F*^2^ are statistically about twice as large as those based on *F*, and *R*- factors based on ALL data will be even larger.

### Fractional atomic coordinates and isotropic or equivalent isotropic displacement parameters (Å^2^)

**Table:**

	*x*	*y*	*z*	*U*_iso_*/*U*_eq_	
O11A	0.46281 (7)	0.8636 (2)	0.32392 (7)	0.0479 (4)	
N4A	0.09143 (8)	0.6731 (3)	0.07459 (9)	0.0423 (4)	
C1A	0.33713 (9)	0.9649 (3)	0.17095 (8)	0.0376 (5)	
C2A	0.32916 (10)	0.7560 (3)	0.13049 (10)	0.0468 (5)	
C3A	0.24921 (10)	0.6600 (3)	0.09931 (10)	0.0458 (5)	
C4A	0.17646 (9)	0.7749 (2)	0.10760 (8)	0.0343 (4)	
C5A	0.18206 (9)	0.9815 (3)	0.14700 (9)	0.0423 (5)	
C6A	0.26212 (10)	1.0747 (3)	0.17881 (10)	0.0435 (5)	
C21A	0.49028 (10)	0.9216 (3)	0.25331 (10)	0.0541 (6)	
C31A	0.42384 (9)	1.0756 (3)	0.20306 (10)	0.0493 (5)	
O11	0.04161 (6)	−0.00501 (19)	−0.08313 (7)	0.0524 (4)	
O12	0.10800 (7)	0.31952 (19)	−0.03468 (7)	0.0518 (4)	
O31	0.42127 (8)	0.3454 (2)	0.01994 (9)	0.0690 (5)	
O32	0.48427 (7)	0.1694 (2)	−0.06214 (8)	0.0596 (4)	
O51	0.34790 (7)	−0.5258 (2)	−0.18874 (7)	0.0600 (4)	
O52	0.21061 (8)	−0.5622 (2)	−0.20625 (7)	0.0544 (4)	
N3	0.42210 (8)	0.2056 (2)	−0.03294 (8)	0.0456 (5)	
N5	0.27785 (8)	−0.4606 (2)	−0.17929 (7)	0.0410 (4)	
C1	0.19176 (8)	0.0264 (2)	−0.07694 (8)	0.0335 (4)	
C2	0.26684 (9)	0.1488 (2)	−0.04825 (8)	0.0359 (5)	
C3	0.34373 (9)	0.0683 (3)	−0.06244 (9)	0.0356 (4)	
C4	0.34961 (9)	−0.1305 (2)	−0.10445 (9)	0.0369 (4)	
C5	0.27433 (9)	−0.2501 (2)	−0.13187 (8)	0.0344 (4)	
C6	0.19600 (9)	−0.1775 (2)	−0.11857 (8)	0.0349 (4)	
C11	0.10697 (9)	0.1224 (3)	−0.06366 (9)	0.0376 (5)	
H2A	0.37840	0.67910	0.12420	0.0560*	
H3A	0.24490	0.51900	0.07300	0.0550*	
H5A	0.13250	1.05830	0.15230	0.0510*	
H6A	0.26570	1.21390	0.20610	0.0520*	
H11A	0.4946 (13)	0.751 (4)	0.3511 (12)	0.077 (7)*	
H21A	0.49730	0.78200	0.22460	0.0650*	
H22A	0.54520	1.00100	0.26650	0.0650*	
H31A	0.44640	1.13040	0.15900	0.0590*	
H32A	0.41540	1.20950	0.23400	0.0590*	
H41A	0.0919 (12)	0.580 (3)	0.0292 (12)	0.069 (6)*	
H42A	0.0494 (13)	0.788 (3)	0.0611 (12)	0.069 (6)*	
H43A	0.0745 (12)	0.566 (3)	0.1140 (12)	0.074 (6)*	
H2	0.26560	0.28400	−0.01970	0.0430*	
H4	0.40170	−0.18130	−0.11380	0.0440*	
H6	0.14660	−0.26430	−0.13730	0.0420*	

### Atomic displacement parameters (Å^2^)

**Table:**

	*U*^11^	*U*^22^	*U*^33^	*U*^12^	*U*^13^	*U*^23^
O11A	0.0444 (6)	0.0510 (7)	0.0489 (7)	0.0053 (5)	0.0116 (5)	0.0101 (6)
N4A	0.0347 (7)	0.0486 (8)	0.0425 (8)	−0.0028 (6)	0.0065 (6)	−0.0058 (7)
C1A	0.0356 (8)	0.0449 (9)	0.0319 (8)	−0.0004 (7)	0.0067 (6)	0.0067 (7)
C2A	0.0335 (8)	0.0548 (10)	0.0541 (10)	0.0072 (7)	0.0141 (7)	−0.0055 (8)
C3A	0.0456 (9)	0.0435 (9)	0.0501 (10)	0.0029 (7)	0.0146 (8)	−0.0114 (8)
C4A	0.0328 (7)	0.0388 (8)	0.0308 (7)	−0.0001 (6)	0.0063 (6)	0.0004 (7)
C5A	0.0322 (8)	0.0431 (9)	0.0501 (10)	0.0093 (7)	0.0058 (7)	−0.0048 (8)
C6A	0.0443 (9)	0.0371 (8)	0.0469 (9)	0.0024 (7)	0.0057 (7)	−0.0059 (7)
C21A	0.0331 (8)	0.0813 (12)	0.0484 (10)	−0.0022 (8)	0.0100 (7)	0.0085 (9)
C31A	0.0380 (8)	0.0643 (11)	0.0455 (9)	−0.0075 (8)	0.0092 (7)	0.0108 (8)
O11	0.0340 (6)	0.0542 (7)	0.0722 (8)	−0.0052 (5)	0.0186 (6)	−0.0177 (6)
O12	0.0423 (6)	0.0502 (7)	0.0635 (8)	0.0051 (5)	0.0130 (5)	−0.0216 (6)
O31	0.0583 (8)	0.0695 (9)	0.0818 (10)	−0.0207 (6)	0.0211 (7)	−0.0324 (8)
O32	0.0366 (6)	0.0669 (8)	0.0808 (9)	−0.0044 (5)	0.0251 (6)	−0.0015 (7)
O51	0.0541 (7)	0.0592 (8)	0.0694 (8)	0.0179 (6)	0.0193 (6)	−0.0151 (6)
O52	0.0585 (7)	0.0490 (7)	0.0551 (8)	−0.0050 (6)	0.0111 (6)	−0.0155 (6)
N3	0.0385 (7)	0.0448 (8)	0.0540 (9)	−0.0055 (6)	0.0113 (6)	−0.0019 (7)
N5	0.0487 (8)	0.0381 (7)	0.0366 (7)	0.0082 (6)	0.0102 (6)	−0.0009 (6)
C1	0.0334 (7)	0.0366 (8)	0.0320 (7)	0.0018 (6)	0.0105 (6)	0.0014 (6)
C2	0.0399 (8)	0.0343 (8)	0.0353 (8)	0.0011 (6)	0.0124 (6)	−0.0031 (6)
C3	0.0325 (7)	0.0389 (8)	0.0361 (8)	−0.0015 (6)	0.0093 (6)	0.0010 (7)
C4	0.0341 (7)	0.0410 (8)	0.0381 (8)	0.0058 (6)	0.0136 (6)	0.0029 (7)
C5	0.0413 (8)	0.0335 (7)	0.0294 (7)	0.0056 (6)	0.0098 (6)	0.0004 (6)
C6	0.0338 (7)	0.0362 (8)	0.0343 (8)	−0.0013 (6)	0.0069 (6)	−0.0019 (7)
C11	0.0349 (8)	0.0430 (9)	0.0356 (8)	0.0020 (7)	0.0096 (6)	−0.0029 (7)

### Geometric parameters (Å, °)

**Table:**

O11A—C21A	1.434 (2)	C5A—C6A	1.384 (2)
O11A—H11A	0.89 (2)	C21A—C31A	1.507 (2)
O11—C11	1.2606 (19)	C2A—H2A	0.9300
O12—C11	1.246 (2)	C3A—H3A	0.9300
O31—N3	1.2277 (19)	C5A—H5A	0.9300
O32—N3	1.2289 (18)	C6A—H6A	0.9300
O51—N5	1.2249 (17)	C21A—H21A	0.9700
O52—N5	1.2221 (18)	C21A—H22A	0.9700
N4A—C4A	1.474 (2)	C31A—H31A	0.9700
N4A—H43A	1.005 (19)	C31A—H32A	0.9700
N4A—H41A	0.958 (19)	C1—C2	1.3859 (19)
N4A—H42A	0.936 (19)	C1—C6	1.3939 (17)
N3—C3	1.474 (2)	C1—C11	1.527 (2)
N5—C5	1.4792 (17)	C2—C3	1.385 (2)
C1A—C6A	1.388 (2)	C3—C4	1.377 (2)
C1A—C2A	1.390 (2)	C4—C5	1.377 (2)
C1A—C31A	1.515 (2)	C5—C6	1.386 (2)
C2A—C3A	1.387 (2)	C2—H2	0.9300
C3A—C4A	1.373 (2)	C4—H4	0.9300
C4A—C5A	1.371 (2)	C6—H6	0.9300
			
C21A—O11A—H11A	112.3 (13)	C1A—C6A—H6A	119.00
C4A—N4A—H43A	110.1 (11)	C31A—C21A—H21A	110.00
H41A—N4A—H42A	109.2 (17)	O11A—C21A—H22A	110.00
H42A—N4A—H43A	108.8 (17)	O11A—C21A—H21A	110.00
C4A—N4A—H42A	111.1 (12)	C31A—C21A—H22A	110.00
H41A—N4A—H43A	105.6 (15)	H21A—C21A—H22A	108.00
C4A—N4A—H41A	111.8 (12)	H31A—C31A—H32A	107.00
O31—N3—O32	124.50 (14)	C1A—C31A—H31A	108.00
O32—N3—C3	117.66 (13)	C1A—C31A—H32A	108.00
O31—N3—C3	117.82 (13)	C21A—C31A—H31A	108.00
O51—N5—O52	123.36 (13)	C21A—C31A—H32A	108.00
O52—N5—C5	118.13 (12)	C2—C1—C6	118.82 (12)
O51—N5—C5	118.50 (12)	C2—C1—C11	118.94 (12)
C2A—C1A—C6A	117.62 (14)	C6—C1—C11	122.23 (12)
C6A—C1A—C31A	120.48 (15)	C1—C2—C3	119.55 (12)
C2A—C1A—C31A	121.87 (14)	N3—C3—C2	118.25 (14)
C1A—C2A—C3A	121.28 (15)	N3—C3—C4	118.85 (13)
C2A—C3A—C4A	119.40 (15)	C2—C3—C4	122.88 (14)
N4A—C4A—C3A	119.50 (13)	C3—C4—C5	116.54 (13)
N4A—C4A—C5A	119.71 (13)	N5—C5—C4	117.97 (12)
C3A—C4A—C5A	120.79 (14)	N5—C5—C6	119.34 (12)
C4A—C5A—C6A	119.46 (15)	C4—C5—C6	122.66 (12)
C1A—C6A—C5A	121.45 (16)	C1—C6—C5	119.53 (12)
O11A—C21A—C31A	109.07 (13)	O11—C11—O12	125.45 (14)
C1A—C31A—C21A	115.58 (14)	O11—C11—C1	117.05 (14)
C3A—C2A—H2A	119.00	O12—C11—C1	117.50 (13)
C1A—C2A—H2A	119.00	C1—C2—H2	120.00
C2A—C3A—H3A	120.00	C3—C2—H2	120.00
C4A—C3A—H3A	120.00	C3—C4—H4	122.00
C4A—C5A—H5A	120.00	C5—C4—H4	122.00
C6A—C5A—H5A	120.00	C1—C6—H6	120.00
C5A—C6A—H6A	119.00	C5—C6—H6	120.00
			
O32—N3—C3—C2	−161.49 (14)	C4A—C5A—C6A—C1A	0.8 (2)
O32—N3—C3—C4	16.9 (2)	O11A—C21A—C31A—C1A	−65.65 (18)
O31—N3—C3—C2	19.7 (2)	C6—C1—C2—C3	0.9 (2)
O31—N3—C3—C4	−161.93 (14)	C11—C1—C2—C3	−177.57 (13)
O51—N5—C5—C4	3.63 (19)	C2—C1—C6—C5	−1.49 (19)
O52—N5—C5—C6	1.02 (18)	C11—C1—C6—C5	176.97 (13)
O51—N5—C5—C6	−178.10 (12)	C2—C1—C11—O11	−172.71 (13)
O52—N5—C5—C4	−177.25 (13)	C2—C1—C11—O12	7.4 (2)
C6A—C1A—C2A—C3A	−0.2 (2)	C6—C1—C11—O11	8.8 (2)
C31A—C1A—C2A—C3A	−178.02 (15)	C6—C1—C11—O12	−171.08 (13)
C2A—C1A—C6A—C5A	−0.6 (2)	C1—C2—C3—N3	178.42 (13)
C31A—C1A—C6A—C5A	177.21 (15)	C1—C2—C3—C4	0.1 (2)
C2A—C1A—C31A—C21A	−51.8 (2)	N3—C3—C4—C5	−178.87 (13)
C6A—C1A—C31A—C21A	130.51 (16)	C2—C3—C4—C5	−0.6 (2)
C1A—C2A—C3A—C4A	0.9 (3)	C3—C4—C5—N5	178.18 (13)
C2A—C3A—C4A—C5A	−0.7 (2)	C3—C4—C5—C6	0.0 (2)
C2A—C3A—C4A—N4A	−179.93 (16)	N5—C5—C6—C1	−177.13 (12)
N4A—C4A—C5A—C6A	179.12 (14)	C4—C5—C6—C1	1.1 (2)
C3A—C4A—C5A—C6A	−0.1 (2)		

### Hydrogen-bond geometry (Å, °)

**Table:**

*D*—H···*A*	*D*—H	H···*A*	*D*···*A*	*D*—H···*A*
O11A—H11A···O11^i^	0.89 (2)	1.88 (2)	2.7569 (16)	168 (2)
N4A—H41A···O12	0.958 (19)	1.924 (19)	2.845 (2)	160.8 (17)
N4A—H42A···O11^ii^	0.936 (19)	2.02 (2)	2.8905 (19)	154.0 (18)
N4A—H42A···O12^ii^	0.936 (19)	2.53 (2)	3.1033 (18)	119.9 (14)
N4A—H43A···O11A^iii^	1.005 (19)	1.783 (19)	2.785 (2)	174.4 (19)
C5A—H5A···O11A^iv^	0.93	2.43	3.317 (2)	161

Symmetry codes: (i) *x*+1/2, −*y*+1/2, *z*+1/2; (ii) −*x*, −*y*+1, −*z*; (iii) −*x*+1/2, *y*−1/2, −*z*+1/2; (iv) −*x*+1/2, *y*+1/2, −*z*+1/2.
